# Enterovirus D68 in a 6-year-old acute flaccid myelitis case in China, 2018: a case report

**DOI:** 10.1186/s12879-020-4829-y

**Published:** 2020-02-11

**Authors:** Xiaoli Wang, Pengfei Zhang, Jie Li, Yanhui Chu, Zheng Li, Yang Yang, Fu Li, Shujuan Cui, Da Huo, Yu Wang, Quanyi Wang

**Affiliations:** 1 Beijing Center for Disease Prevention and Control, Beijing Research Center for Preventive Medicine, No.16 Hepingli Middle Street, Dongcheng District, Beijing, 100013 China; 2 Beijing Children’s Hospital, Capital Medical University, National Center for Children’s Health, No.56 Nanlishi Road, Xicheng District, Beijing, 100045 China; 3 Xicheng District Center for Disease Control and Prevention, No.38 De Shengmenwai Street, Xicheng District, Beijing, 100120 China; 4 Institute for Infectious Disease and Endemic Disease Control, Beijing Center for Disease Prevention and Control, No.16 Hepingli Middle Street, Dongcheng District, Beijing, 100013 China

**Keywords:** Enterovirus D68, Acute flaccid myelitis, China

## Abstract

**Background:**

Acute flaccid myelitis (AFM) are reported to be associated with enterovirus D68 infection. Though an increasing number of AFM cases were reported with EV-D68 infection in the US, few such cases have been found in China.

**Case presentation:**

A 6-year-old boy presented with acute flaccid myelitis (AFM) involving left arm after fever and respiratory symptoms for 6 days. Computed Tomography (CT) revealed inflammation in both lungs and magnetic resonance imaging (MRI) of the brain and spine showed swelling in the left frontal lobe and brain stem. The patient was diagnosed with meningomyelitis. EV-D68 was detected from pharyngeal samples 36 days after the onset of the disease.

**Conclusion:**

We report the first EV-D68 infection in case of AFM in mainland China. AFM surveillance systems is recommended to be established in China to guide diagnosis, case reporting, and specimen collection and testing for better understanding its etiologies.

## Background

Enterovirus D68 (EV-D68), mostly associated with severe respiratory tract infections in children, has emerged in the United States in 2014, followed by Canada, Europe and Asia [1–4]. In addition to respiratory illness, some studies showed that EV-D68 infection was associated with neurological complications, mainly acute flaccid myelitis (AFM) [5], which has raised a new public health concern. Though an increasing number of AFM cases were reported with EV-D68 infection in the US, few such cases have been found in China. In this study, we present the first enterovirus 68 infection in a 6-year-old AFM case in mainland China.

## Case presentation

A previously healthy 6-year-old boy, fully immunized with routinely recommended vaccines from Heze City, Shandong Province, complained of a sore throat and cough on September 21, 2018. He experienced high fever (38.5 °C) 3 days later and increased to 39.6 °C with vomiting on September 25. Intravenous antibiotics were administered at local hospital from Heze. Left arm weakness was noted on day 6 and then progressed to his right arm and both legs over the next 2 days which made the patient unable to walk without help.

On Computed Tomography (CT) of the chest, inflammation in both lungs was observed, and then intravenous immunoglobulin (IVIG) was administered. Magnetic resonance imaging (MRI) of the brain and spine on day 9 showed swelling in the left frontal lobe and brain stem and spinal cord lesions in the grey matter and ventricornu at the cervical and thoracic levels. The patient was diagnosed with meningomyelitis. On day 10, he became dyspneic and was intubated for assisted ventilation. Acyclovir was administered and pulse steroid therapy and mannitol were prescribed. Cerebral spinal fluid (CSF) data showed pleocytosis (259 × 10^6^ cells/L, 93% mononuclear). The CSF glucose level was 5.02 mmol/L (norm:2.8–4.5), CSF protein level was 380 mg/ L(norm:20–450) and CSF chloride level was 134.0 mmol/L (norm:118–129); *cryptococcusneoformans* and acid-fast bacillus were tested negative. Serological analysis showed no evidence of Epstein-Barr (EB) virus, cytomegalovirus, parvovirus B19, human herpesvirus 6, rubella virus, herpes simplex virus, or coxsackievirus infection. On day 18, an MRI revealed lesions in brain and at the C1-C7 levels.

**Fig. 1:**
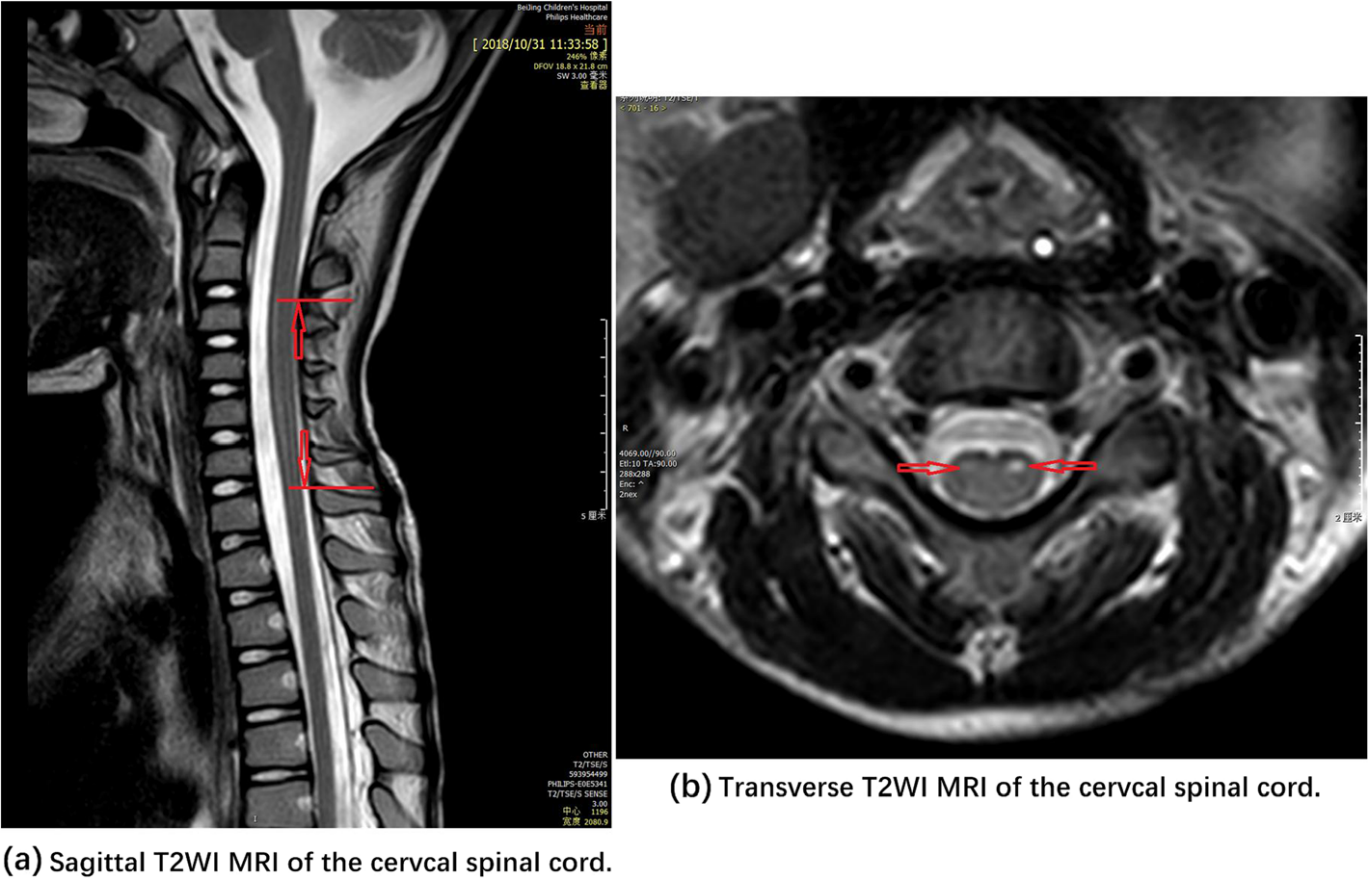
Sagittal and Transverse T2WI MRI of the cervical spinal cord. *Note: Arrows show abnormal signals of vertricornu over C2-C6. MRI = magnetic resonance imaging, T2WI = T2-weighted image

With no improvement in his neurologic deficits, he was transferred to Beijing Children’s Hospital, an affiliated hospital of the Capital Medical University of China on October 24. At admission, physical examination revealed that blood pressure, pulse, and temperature were normal. Coarse breath sound and scattered moist rales could be heard over both lung fields. The muscle power scale of the right arm, left arm and both legs were grades 1, 0 and 4, respectively, based on the Medical Research Council (MRC) Score. Abdominal and deep tendon reflexes could be elicited. The Babinski sign was negative, while neck stiffness was equivocally positive (resolved on November 1). Muscle tension was hypotonic in both upper arms and normal in his lower arms. A red macula papular rash with peeling was observed on both palms. MRI of the brain and spine on October 31 revealed abnormal signal of ventricornu of cervical spinal cord at the C2-C6 levels, prompting the injury of the motor nerve cells and pineal cyst in brain (Fig. 1). Methylprednisolone was then administered and decremented starting on October 31. A tracheotomy was performed in consideration for long-term ventilatory support. Nerve growth factor (NGF) and vitamins B were initiated for nutrition of impaired nerve cells. He was transferred to another children’s hospital on November 20 for neurorehabilitation.

On December 17, after one-month of physical therapy, he could walk with assistance and there was significant improvement in the distal right upper arm strength, increasing from grade 0 to grade 3. Motor impairment was worse in the left arm upper arms, the muscle power scale were grade 0. The muscle power scales of both legs were grades 5. By December 31, he could walk steadily. He was able to breathe without mechanical ventilation during daytime.

**Fig. 2:**
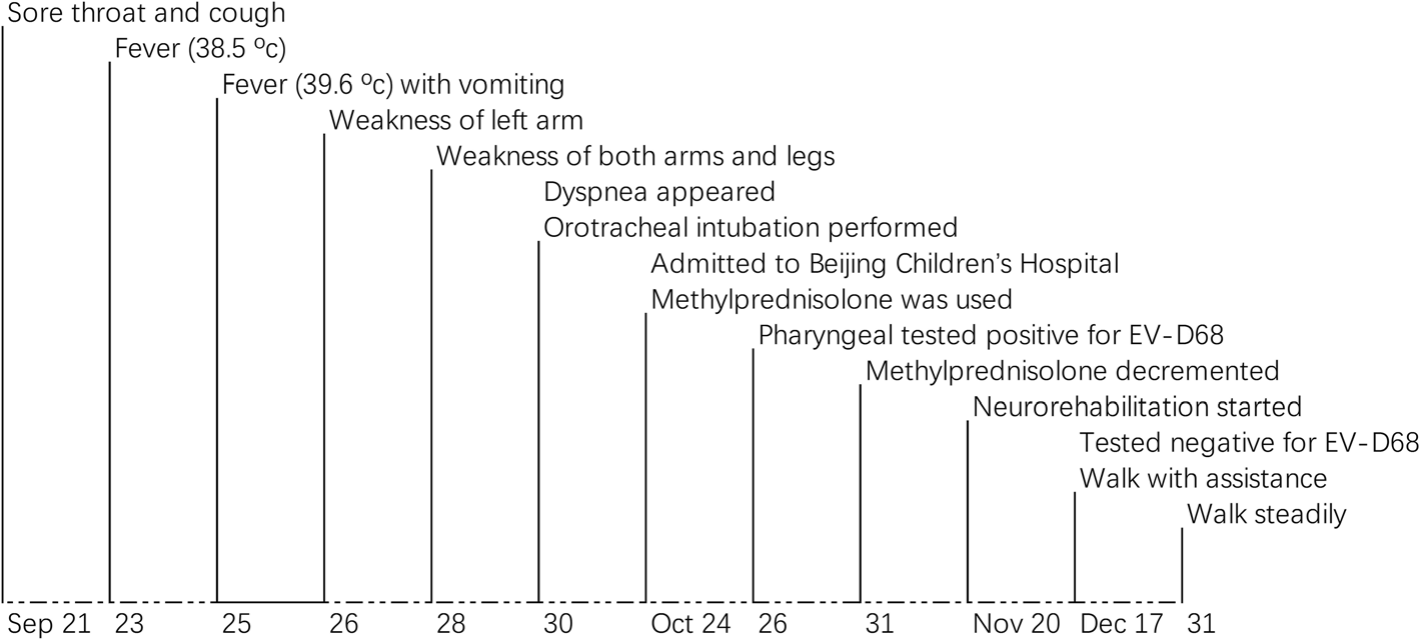
Timeline of clinical and laboratory events

Pharyngeal and blood specimens were collected on day 36. Specimens were stored in minimum essential medium (MEM) and transferred to Beijing Center for Disease Prevention and Control (CDC) for laboratory analyses. Following reports from the United States on an increasing number of EV-D68 associated AFM cases, a total nucleic acid extraction was performed and was tested for EV-D68, polio, enterovirus 71 (EV-A71), and coxsackievirus A16 (CV-A16) with real-time reverse transcriptase polymerase chain reaction (RT-PCR) (Jiangsu BioPerfectus Technologies Co., Ltd., China) which targeted the partial VP1 gene for EV-D68, EV-A71, CV-A16 and targeted the polyprotein gene for polio. The pharyngeal specimen were tested positive for EV-D68 with the [cycle threshold (Ct) value of 33], and negative for polio, EV-A71, and CV-A16. In order to investigate the phylogenetic features of this virus, next-generation sequencing were performed. We conducted deep sequencing of EV-D68 and found two identical reads to the prototype EV-D68 strain (NC_038308.1). One read (29 bp, from 4390 to 4418, 3 × sequencing depth) was on 2C region of EV-D68, the other one (41 bp, from 7235 to 7275, 6 × sequencing depth) was on 3D region of EV-D68. We tried to amplify the complete VP1 sequence of EV-D68 by using conventional RT-PCR but did not succeed. Stool and blood specimens collected on December 17 were tested negative for EV-D68. Figure 2 showed a timeline of clinical and laboratory events.

His parents reported that he had no history of travel during the 2 weeks before illness onset and no family history.

## Discussion and conclusions

Since the EV-D68 outbreak reported by the United States Centers for Disease Control and Prevention (CDC) in 2014, EV-D68 has been increasingly associated with acute flaccid myelitis in other researches [5–13]. Previous studies in 2014 showed that the odds of EV-D68 infection were 10.3 times higher for children with AFM than for respiratory pathogen panel (RPP)-tested controls [14].

In mainland China, some provinces had been carried out EV-D68 tests among acute flaccid paralysis (AFP) cases, however, no AFP case with EV-D68 infection has been reported in China, except in Taiwan [15]. The clinical case criteria for AFM has not been defined in China yet. According the case definitions in US, this 6-year-old case meet the confirmed AFM case definition [12]. This is the first EV-D68 infection detected in case of AFM in mainland China. Intrathecal EV-D68 antibody detection or viral detection in cerebrospinal fluid could be confirmatory of EV-D68 being the neurotrophic pathogen. The detection assays were not performed since CSF was not collected in Beijing Children’s Hospital. EV-D68 has been detected in the patient’s pharyngeal 36 days after the onset of the disease, which led to the hypothesis that the EV-D68 could persist for at least a month. However, the possibility of secondary infection at the late stage cannot be ruled out. It is of clinically importance to elucidate the EV-D68 infection procedure evidenced by seroconversion. Unfortunately, the serum sample at the early onset of the case was unavailable due to his complicated transference experience. Considering the serious clinical outcomes, AFM surveillance systems should be established in China to guide diagnosis, case reporting, and specimen collection and testing. Such guidelines can help identify additional AFM cases and determine their etiologies.

## Data Availability

The datasets used and/or analyzed during the current study are available from the corresponding author on reasonable request.

## Abbreviations

AFM: Acute flaccid myelitis
AFP: Acute flaccid paralysis.
CDC: Center for Disease Prevention and Control
CSF: Cerebral spinal fluid
CT: Computed tomography
CV-A16: Coxsackievirus A16
EV-A71: EnterovirusA71
EV-D68: Enterovirus D68
IVIG: Intravenous immunoglobulin
MEM`: Minimum essential medium
MRI: Magnetic resonance imaging
NGF: Nerve growth factor
NGS: Next-generation sequencing
RPP: Respiratory pathogen panel
RT-PCR: Reverse transcriptase polymerase chain reaction
